# Editorial: Relevance of Steroid Biosynthesis, Metabolism and Transport in Pathophysiology and Drug Discovery

**DOI:** 10.3389/fphar.2019.00245

**Published:** 2019-03-26

**Authors:** Tea Lanišnik Rižner

**Affiliations:** Faculty of Medicine, Institute of Biochemistry, University of Ljubljana, Ljubljana, Slovenia

**Keywords:** intracrine action, steroid hormones, transporters, sulfatase, cancer

Steroids have crucial roles in human physiology, from prenatal sexual differentiation, through puberty and adulthood, to menopause/andropause and old age. Through binding corresponding receptors, steroids exert endocrine, paracrine, and intracrine actions. In peripheral tissues, active steroid levels depend on their plasma concentrations and local formation from their precursors, dehydroepiandrosterone-sulfate (DHEA-S), and estrone sulfate (E1-S). Steroid sulfates require transporters of the organic-anion-transporting polypeptide (OATP) and organic-anion-transporter (OAT) families for uptake, and ATP-binding cassette (ABC) pumps for removal. Any dysregulation of uptake of steroid precursors, steroid hormone activation, actions, metabolism, or excretion can lead to development of malignant and benign diseases. This Research Topic aims to provide an up-to-date view of steroid transport, metabolism, and actions, with emphasis on involvement of these processes in pathophysiology, and on associated drug targets and novel steroidal compounds as leads for potential novel therapeutics. There are four comprehensive reviews on intracrine steroid actions, followed by five research papers on transport, biosynthesis, and metabolism of steroid hormones, and, finally, two papers on new steroidal lead compounds with anti-proliferative actions.

The detailed review by Konings et al. focuses on steroidogenesis in ovaries and peripheral tissues, and describes the actions of steroid hormones in the endometrium, gastrointestinal tract, bone, lungs, central nervous system, adipose tissue, and immune system, and their associations with disease. Approved drugs that target intracrine enzymes are discussed, along with novel therapeutic approaches. The authors emphasize the need for further validation studies, and recommend development of dual/ triple inhibitors.

Manuscript by Chatuphonprasert et al. deals with steroid biosynthesis, transport, and metabolism in human placenta. The authors describe steroids that have crucial roles in pregnancy and embryo development, “cholesterol has roles *per se* and as a precursor of steroid hormones,” and its uptake, intracellular transport, and efflux are summarized. Progesterone and estrogen synthesis in placenta and glucocorticoid synthesis in fetal organs, and the interplay between these organs, are also described.

Also neurosteroids, DHEA-S and Pregnenolone-sulfate need transporters to cross cell membranes. The mini review by Grube et al. discusses the published data on the ABC and solute carrier (SLC) transporters, putatively involved in secretion of DHEA-S and pregnenolone sulfate from neurons and glial cells, and their transport through the blood-brain and blood-cerebrospinal fluid barriers. They emphasize that the functions of ABC and SLC transporters in the brain remain poorly understood.

Estrogen-dependent malignancies predominantly affect post-menopausal women and depend on local formation of estrogens from DHEA-S and E1-S. The intracrine actions of estrogens in endometrial and ovarian cancers are reviewed by Rižner et al. The authors focus on DHEA-S and E1-S transporters and intracrine enzymes, and their dysregulated expression in gynecological cancers. Steroid sulfatase, 17-keto-steroid reductase type 1, estrogen receptors, and the individual OAT, OATP, and ABC transporters are discussed as potential new pharmacological targets, and difficulties associated with these approaches are considered.

The first research paper confirms the importance of the sulfatase pathway for estrogen formation in endometrial cancer. Sinreih et al. report that estradiol (E2) is formed only from E1-S and E1, and not from androstenedione, with increased E2 levels in cancer compared to adjacent control tissue. The key genes of the aromatase and sulfatase pathways are not differentially expressed, but immunohistochemistry reveals intense staining for sulfatase and weak staining for sulfotransferase SULT1E1, supporting the prevalence of the sulfatase pathway over the aromatase pathway.

The clinical significance of OATP and ABC transporters in high-grade serous ovarian cancer, the most frequent and aggressive subtype of ovarian cancer, was investigated by Svoboda et al. The *SLCO5A1* gene, encoding OATP5A1 and implicated in transport of estradiol glucuronides, was identified as an independent positive prognostic factor for overall survival. However, as the authors conclude, further validation studies on larger collections of high-grade serous ovarian cancer are needed.

Also in breast cancer, E1-S acts as a source of E2. As OATP and OAT transporters have already been studied, Karakus et al. focused on the sodium-dependent organic anion transporter (SOAT). They confirm SOAT expression in different breast pathologies and its role in E1-S uptake in stably transfected T47D-SOAT cells. E1-S more efficiently stimulates proliferation of T47D-SOAT cells compared to control cells, while a SOAT inhibitor blocks E1-S stimulation, which supports the role of SOAT in E1-S uptake and estrogen action.

The epidemiological studies disclosed that hormone replacement therapy that includes E1-S and progestins reduces the risk of colorectal cancer in post-menopausal women. Gilligan et al. thus inspected the actions of E1-S in model cell lines of colorectal cancer. After translocation, apparently *via* OATP4A1, E1-S is metabolized to active estrogens, and stimulates GPER. Surprisingly, GPER agonists increase steroid sulfatase activity. The authors conclude that hormone replacement therapy “may play a dual role in the incidence and progression of colorectal cancer,” where “tamoxifen, and fulvestrant may negatively impact colorectal cancer patients outcome.”

In patients with breast cancer, epidemiological studies have indicated beneficial effects of isoflavones only when ERα-negative, and not when ERα-positive. This led Poschner et al. to investigate the effects of genistein and daidzein on estrogen conjugation in ERα-positive MCF-7 breast cancer cells. Both of these isoflavones stimulate cell proliferation and inhibit estrogen conjugation, especially sulfation, and less glucuronidation. As the authors indicate, these effects of isoflavones would be expected only in patients after consumption of high-dose supplements.

Steroidal compounds are used for treatment of several gynecological conditions and hormone-dependent forms of cancer. For new avenues of treatment, additional compounds with anti-proliferative properties are needed. Gyovai et al. investigated five novel 19-nortestosterone analogs. The most potent, the 17α-chloro derivative, showed moderate cytotoxic effects, induced apoptosis, stabilized microtubule formation, and showed negligible androgenic activity, and thus exemplifies “an excellent skeleton for designing novel anti-proliferative steroidal agents.” The last paper, by Scherbakov et al., reports on eight novel steroidal pyrimidines and dihydrotriazines. Their lead compound, a 16-C dihydrotriazine-modified estrane, displays greater cytotoxicity toward ERα-positive MCF-7 cells compared to ERα-negative MDA-MB231 cells, and partially down-regulates expression of ERα, which suggests its potential for design of novel SERM.

The associations between steroid hormones and pathophysiological processes are still not completely understood, and in particular, membrane transport of steroid precursors and metabolites has not had sufficient attention to date. This Research Topic thus fills in some of the gaps in our knowledge, and provides novel information on steroid transporters in peripheral tissues and ovarian, endometrial, breast, and colorectal cancers, and substantiates the impact of the sulfatase pathway for E2 action in these pathologies. Also new data on the mechanisms of isoflavone actions and on lead compounds with anti-proliferative effects are provided. However, there is still a great deal more to be discovered on the importance of steroid biosynthesis, metabolism and transport in disease, which warrants further studies.

## Author Contributions

The author confirms being the sole contributor of this work and has approved it for publication.

### Conflict of Interest Statement

The author declares that the research was conducted in the absence of any commercial or financial relationships that could be construed as a potential conflict of interest.

